# Complete genome sequence of strain M4-SHS-6, a potential new member of the genus *Acidithiobacillus*

**DOI:** 10.1128/mra.00152-25

**Published:** 2025-05-27

**Authors:** Minjie Zhou, Tiantian Yu, Xiaomin Lan, Sidong Zhu, Jigang Chen

**Affiliations:** 1 College of Biological and Environmental Sciences, Zhejiang Wanli University 12463, Ningbo, Zhejiang, China; University of Southern California, Los Angeles, California, USA

**Keywords:** sulfur-oxidizing bacteria, *Acidithiobacillus*, genome sequencing

## Abstract

Here, we report the complete genome sequence of strain M4-SHS-6, a potential chemolithoautotrophic sulfur-oxidizing bacterium. The genome comprises one circular chromosome and two circular plasmids, assembled through hybrid sequencing (Illumina and Nanopore).

## ANNOUNCEMENT

Strain M4-SHS-6 was isolated from a surface water sample collected in April 2018 from an artificial lake at Zhejiang Wanli University, China (GPS: 121.6°E, 29.8°N). A 100 µL aliquot of the sample was enriched in 10 mL of liquid DSMZ medium 68 (pH 6.5) containing Na_2_S_2_O_3_ as the sole energy source. The enrichment culture exhibiting a significant pH decrease was transferred to DSMZ medium 68-agar via dilution plating. A colony (designated M4-SHS-6) showing a yellow halo zone on DSMZ medium 68-agar plates after 7 days of incubation at 30°C was purified through repeated streaking.

Colony PCR targeting the nearly full-length 16S rRNA gene was performed using universal bacterial primers 27F and 1492R (1). The 16S rRNA sequence was manually validated and deposited in GenBank (accession no. PQ637443). EzTaxon-e analysis (2) indicated that strain M4-SHS-6 shares 94.2%–98.4% sequence similarity with type strains of the genus *Acidithiobacillus*, with the closest relative being *Acidithiobacillus ferridurans* ATCC 33020^T^ (98.4% identity; GenBank accession no. AJ278719).

Genomic DNA extracted from exponential phase cells (CTAB method(3)) was used for Illumina and Oxford Nanopore sequencing. The Illumina library was prepared using the NEXTflex Rapid DNA-Seq Kit (Bioo Scientific, USA) and sequenced on the Illumina HiSeq X Ten platform (Illumina Inc., USA). Approximately 1.46 Gb of raw data of 150-bp-long paired-end reads was generated. A total of 4,837,194 reads were subjected to control and trimming using SOAPnuke version 1.3.0 (4), generating a total of 4,811,505 clean reads. For Nanopore sequencing, DNA was sheared using g-TUBE, and large DNA fragments (>20 Kb) were selected using BluPippin (Sage Science, Beverly, MA, USA). The Nanopore library was prepared using the SQK-LSK109 kit (Oxford Nanopore Technologies, UK) and loaded into an R9.4.1 flow cell for the PromethION platform (Oxford Nanopore Technologies, UK). The base calling was performed using Guppy version 4.0.15 (Oxford Nanopore Technologies). Read QC was achieved using NanoPlot version 1.15.0 with a threshold value (*Q*) of >7 (5). Nanopore sequencing yielded 251,745 clean reads, with an average length of 5,660 bp and an *N*_50_ value of 8,791 bp.

Hybrid assembly of both read types was performed using Unicycler version 0.4.8 (6) and resulted in a single circular chromosome (3,003,844 bp) and two circular plasmids, pLA (8,964 bp) and pLB (3,693 bp), with an overall G + C content of 56.1%. We stated that pLA and pLB are plasmids verified by Plasflow (7) and PLSDB database (8). Genome coverage reached 99.68%, with average depths of 482.6× (Illumina) and 472.4× (Nanopore). For all software, default parameters were used, except where otherwise noted. Genome annotation via NCBI PGAP (9) predicted 3,144 genes, including 2,993 protein-coding sequences. The average nucleotide identity between M4-SHS-6 and *Acidithiobacillus* type strains ranged from 70.1% to 80.5%, below the interspecies threshold (95.0% (10)).

## Data Availability

This whole-genome sequence has been deposited in GenBank under accession numbers CP175559 (chromosome), CP175560 (pLA), and CP175561 (pLB). The BioProject and BioSample accession numbers are PRJNA1190523 and SAMN45048708, respectively. Raw sequencing data are accessible via SRA entries SRR32044594 (Illumina) and SRR32044593 (Nanopore).
